# Is there a relationship between middle concha bullosa and ethmoid roof asymmetry?

**DOI:** 10.1016/j.bjorl.2020.06.003

**Published:** 2020-07-19

**Authors:** Resit Murat Acikalin, Ozlem Bayram, Cemal Haci, Huseyin Tarik Yanik, Yusuf Ozturkcu, Ayhan Kocak, Aykut Insan

**Affiliations:** a Haseki Training and Research Hospital, Department of Otorhinolaryngology, Istanbul, Turkey; b Acibadem Healthcare Group, Taksim Hospital, Department of Otorhinolaryngology, Istanbul, Turkey; c Haseki Training and Research Hospital, Department of Radiology , Istanbul, Turkey

**Keywords:** Ethmoid roof, Concha bullosa, Asymmetry

## Abstract

**Introduction:**

The middle turbinate and ethmoid roof are intranasal structures and may have many anatomical variations. These structures, which serve as anatomical markers during functional sinus surgery, are important for preventing complications and performing a proper surgery. Knowledge of anatomical variations will increase surgical success and reduce complications.

**Objective:**

We aimed to investigate the presence of asymmetry in the ethmoidal roof and anatomical variation in patients with and without concha bullosa.

**Methods:**

In this study, the files of patients who underwent paranasal computed tomography between 2012 and 2018 were analyzed retrospectively. The patients were divided into two groups, as patients with and without concha bullosa. Differences between the two groups in terms of age, gender, septum deviation, ethmoid artery dehiscence, ethmoid roof asymmetry were examined.

**Results:**

The 369 patients included in our study were divided into two groups; those with concha bullosa and those without concha bullosa. The mean age of the patients with concha bullosa was 36.1 ± 13.4 (min–max: 12–74) and the mean age of patients without concha bullosa was 37.5 ± 14.3 (min–max: 10–81). The ethmoid roof depths were compared between the two groups and a significant difference was observed (*p* < 0.001). The ethmoid roof depth was higher in the group with concha bullosa (*p* < 0.001).

**Conclusion:**

The results of our study indicate that the ethmoidal roof tends to be higher in patients with middle concha bullosa.

## Introduction

Many anatomical variations related to the middle turbinate have been described. The most common of these is the concha bullosa. Formation mechanisms of concha bullosa and the factors affecting concha bullosa are unclear. However, if the concha bullosa is large enough, it may obstruct the nasal passage and affect breathing and odor function adversely.^1^

The ethmoid roof is formed by the fovea ethmoidalis, which is part of the orbital extension of the frontal bone. This structure separates the ethmoid air cells from the anterior cranial fossa. The fovea ethmoidalis articulates with the lateral lamella of the cribriform layer of the ethmoid bone medially.^2^ Asymmetries are sometimes seen in the anterior skull base and ethmoid roof, and these abnormalities are important for avoiding intracranial complications during endoscopic sinus surgery. Endoscopic concha bullosa surgery is relatively easy to perform and is very useful in the appropriate patient group, but it is important to know the anatomic variations to avoid complications.^3^

In this study, in order to evaluate the relationship between concha bullosa and ethmoid roof asymmetry, we examined patients who underwent paranasal CT before endonasal surgery and investigated whether there was a significant difference between the right and left ethmoid roof depths in patients with unilateral concha bullosa and other anatomical variations (septum deviation, vascular dehiscence etc.)

## Methods

In this study, files of patients who underwent paranasal sinus computed tomography in our institution between 2012 and 2018 were examined retrospectively. Computed tomography results of the patients were evaluated and recorded by an otorhinolaryngologist and radiologist at the same time. Ethical approval of the present study was obtained from the ethics committee of an education and research hospital (2019–19/22).

The number of patients included in the study was 369. The patients included in our study were divided into two groups as unilateral concha bullosa and non-concha bullosa.

As an exclusion criterion, patients under the age of 18, with nasal polyposis, bilateral concha bullosa, malformation previously caused by head trauma who who have previously had sinus surgery, were excluded from the study.

**Figure 1:**
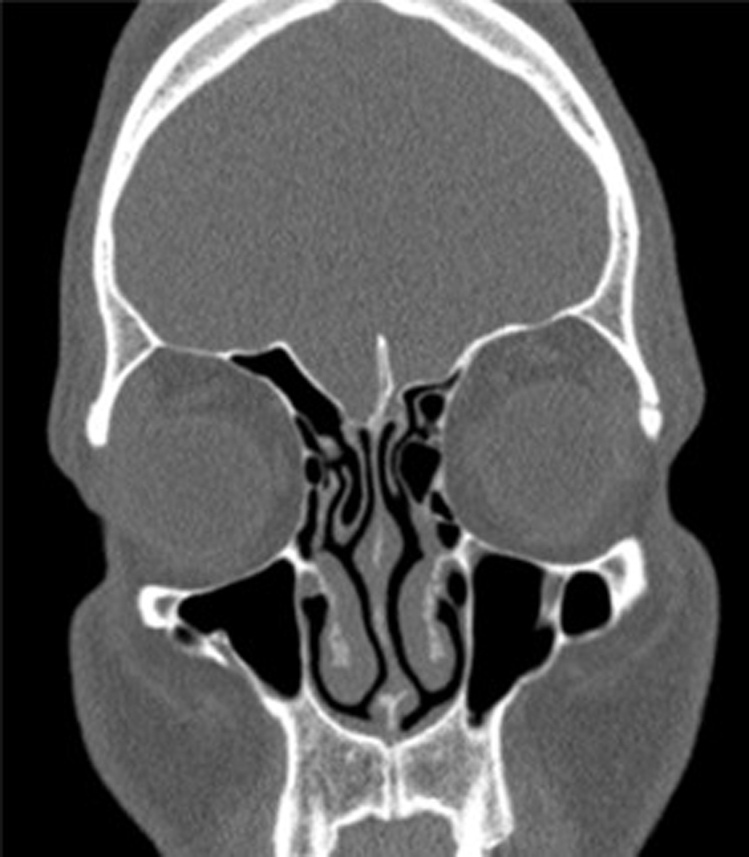
Etmoid roof asymmetry and concha bullosa. Unilateral concha bullosa and ethmoid roof asymmetry are evident. Coronal section the paranasal sinus CT image is the section where the infraorbital nerve was first seen, and all measurements were taken from this section.

Age, gender, physical examination findings, and right and left ethmoid roof depths of the patients were measured. In order to evaluate the roof asymmetry of the patients, measurements were made by comparing the right and left coronal plane and calculating the distance. The depth of the lateral coverslip was calculated by subtracting the depth of the cribriform plate from the depth of the medial roof (Fig. 1). The measurements of our patients were taken from the first section of the infraorbital nerve from the coronal CT images, and a comparison was made between the groups.

The relationship between the roof depths of the patients with and without concha bullousa was evaluated.

In this study, it was investigated whether there is a relationship between concha bullosa and the ethmoid roof.

### Statistical method

SPSS 15.0 program for Windows program was used for statistical analysis. Descriptive statistics: the number and percentage for categorical variables, mean, standard deviation, minimum, maximum for numerical variables were given. Mann Whitney *U* test was used for the comparison of numerical variables in two independent groups, since no normal distribution condition was provided. The ratios were compared with Chi-Square analysis. Statistical alpha significance level was accepted as *p* < 0.05.

## Results

The 369 patients included in the study were divided into two groups as patients with concha bullosa and without concha bullosa. The number of patients with concha bullosa was 193 (109 females, 84 males) and the number of patients without concha bullosa was 176 (81 females, 95 males). The mean age of the patients with concha bullosa was 36.1 ± 13.4 (min–max: 12–74) and the mean age of patients without concha bullosa was 37.5 ± 14.3 (min–max: 10–81).

The right ethmoid roof depth of the group with concha bullosa was 5.25 ± 1.78 mm (min–max: 1.5–12.3 mm), the left ethmoid roof depth with concha bullosa was 5.31 ± 1.95 mm (min–max: 1.5–17 mm), the right ethmoid roof depth of the group without concha bullosa 5.94 ± 1.75 mm (min–max: 1.9–11 mm), left ethmoid roof depth mean 5.93 ± 1.75 mm (min–max: 2.1–11.6 mm). When the ethmoid roof depths were compared between the two groups, a significant difference was observed (*p* < 0.001).

In the same group and same patients, the differences between the right and left ethmoid roof depths were measured and a significant difference was observed as compared with the group with and without concha bullosa. There was a significant difference between the group with concha bullosa and the group without concha bullosa in terms of roof asymmetry (*p* < 0.001) (Table 1).

**Table 1:** Ethmoid roof asymmetry in patients with concha bullosa and without concha bullosa.

	With concha bullosa		Without concha bullosa		
	Mean ± SD (mm)	Min–max (Median) (mm)	Mean ± SD (mm)	Min–max (Median) (mm)	
The right ethmoid roof depth	5.25 ± 1.78	1.5–12,3 (5.1)	5.94 ± 1.75	1.9–11 (5.8)	<0.001*
The right ethmoid roof depth	5.31 ± 1.95	1.5–17 (5.4)	5.93 ± 1.75	2,1–11,6 (5.9)	<0.001*
The ethmoid roof depth difference	1.51 ± 0.81	0–5.4 (1.2)	0.89 ± 0.93	0–4.9 (0.6)	<0.001*

The etmoid roof	n	%	n	%	*p*
Symetric	12	6.2	107	60.8	<0.001*
Asymetric	181	93.8	69	39.2	

* indicates statistically significant values.

When septal deviation and anterior ethmoid artery dehiscence were compared between groups with and without concha bullosa, a significant difference was found between the two groups in terms of septum deviation (*p* < 0.001), but there was no difference between anterior ethmoid artery dehiscence (*p* = 0.127). Septum deviation was more frequent in patients with concha bullosa.

## Discussion

During endoscopic sinus surgery, the surgeon should consider these variations when planning the operation and decide the extent of the surgery. The ethmoidal roof is the upper limit of endoscopic sinus surgery.^3^ In order to prevent complications, paranasal computed tomography of the patients should be carefully examined and if there is a variation related to this area, it should be paid attention to during surgery.^4^, ^5^, ^6^

One of the most common variations of the ethmoid roof is ethmoid roof asymmetry. In this case, the length of the lateral lamellae, which is one of the structures constituting the fovea ethmoidalis, differs from each other on the right and left sides. Consequently, depth differences and form differences may be seen in fovea ethmoidalis. If these differences are not noticed the likelihood of injury to the anterior skull base increases.^2^

Concha bullosa is a common variation in the community due to aeration of the middle turbinate. Although the frequency varies in many studies, it is encountered in between approximately 10% and 50% of patients.^7^, ^8^ The presence of concha bullosa can lead to various variations in the nose and ethmoid labyrinth. The middle concha bullosa may be unilateral or bilateral. In one study, it was reported that septal deviation increased significantly in patients with unilateral concha bullosa.^9^ In the present study, septum deviation was observed more frequently in patients with concha bullosa.

In a study conducted by Apuhan et al., 45% of the patients with concha bullosa were female and 55% were male.^10^ In our study, concha bullosa was seen in 43% of male patients and 57% of female patients. In a study conducted by Cheng et al., the mean age of patients with concha bullosa was 46.7 years, whereas in the present study it was 36.1.^11^ Concha bullosa is a variation that can be seen at any age after the development of the ethmoid labyrinth.

The most common sideedness of concha bullosa has been investigated in many studies, but there is no clear opinion on this issue. In a study conducted by Hatipoğlu et al., 47% of patients with concha bullosa were found to be bullous concha on the right side and 44% on the left.^12^ In another study conducted on patients with concha bullosa, 41% were seen on the right and 39.1% on the left side.^13^ In the present study, in patients with concha bullosa, 42.5% of the concha bullosa patients were on the right side and 57.5% on the left.

One of the most serious complications of endoscopic sinus surgery is anterior ethmoid artery injury. This risk increases especially in patients with anterior ethmoid artery dehiscence. Therefore, many studies have investigated anatomical variations that may be related to this phenomenon. However, there are no studies in the literature to reveal a possible relationship. In a study conducted by Stammberger and Posawetz the relationship between anterior ethmoid artery dehiscence and the agger nasi cell and frontal cells was investigated, but there was no significant relationship.^14^ In this study, the relationship between concha bullosa and anterior ethmoid artery dehiscence was investigated and there was no difference between patients with and without concha bullosa.

Since concha bullosa is a variation due to aeration of the middle turbinate, it also affects the development of anatomical structures close to it. According to the theory of ex vacuo in patients with septum deviation, the concave side of the nasal cavity can trigger a concha ventilation and cause concha bullosa.^15^ Gün et al. found a significant relationship between the axial diameter of the concha bullosa and the width of the anterior ethmoid roof. However, the same relationship could not be determined with respect to the vertical diameter of the middle turbinate. These findings suggest that the position of the perpendicular lamina changes due to the aeration of the middle turbinate. Variations in the ethmoidal roof are also likely to be due to changes in the perpendicular laminate. In this study, the relationship between unilateral concha bullosa and ethmoid roof asymmetry was investigated in order to test this possible hypothesis. As shown in Table 1, the rates of ethmoid roof asymmetry in patients with and without concha bullosa were compared. As a result, the rate of ethmoid roof asymmetry in patients with unilateral concha bullosa was significantly higher than in patients without concha bullosa (*p* < 0.001). Aydoğan et al. in a study on ethmoidal roof asymmetry examined the most common anatomic variations in patients while investigating ethmoid roof and 81.8% of patients demonstrated nasal septal deviation and concha bullosa in 30%.^16^ In this study, septum deviation was more frequent in patients with concha bullosa in accordance with the literature.

## Conclusion

The results of our study indicate that the ethmoidal roof tends to be higher in patients with middle concha bullosa.

## Conflicts of interest

The authors declare no conflicts of interest.
